# The Gut Microbiome in Myalgic Encephalomyelitis (ME)/Chronic Fatigue Syndrome (CFS)

**DOI:** 10.3389/fimmu.2021.628741

**Published:** 2022-01-03

**Authors:** Rahel S. König, Werner C. Albrich, Christian R. Kahlert, Lina Samira Bahr, Ulrike Löber, Pietro Vernazza, Carmen Scheibenbogen, Sofia K. Forslund

**Affiliations:** ^1^ Faculty of Medicine, University of Basel, Basel, Switzerland; ^2^ Division of Infectious Diseases and Hospital Epidemiology, Cantonal Hospital St. Gallen, St. Gallen, Switzerland; ^3^ Division of Infectious Diseases and Hospital Epidemiology, Children’s Hospital of Eastern Switzerland, St. Gallen, Switzerland; ^4^ Charité - Universitätsmedizin Berlin, Freie Universität Berlin and Humboldt-Universität zu Berlin, Berlin, Germany; ^5^ Experimental and Clinical Research Center, A Joint Cooperation of Max-Delbrück Center for Molecular Medicine and Charité-Universitätsmedizin Berlin, Berlin, Germany; ^6^ Host-Microbiome Factors in Cardiovascular Disease, Max Delbruck Center for Molecular Medicine in the Helmholtz Association (MDC), Berlin, Germany; ^7^ Institute for Medical Immunology, Charité-Universitätsmedizin Berlin, Berlin, Germany; ^8^ European Molecular Biology Laboratory, Structural and Computational Biology Unit, Heidelberg, Germany

**Keywords:** ME/CFS, Chronic Fatigue Syndrome (CFS), Myalgic Encephalomyelitis (ME), microbiome, gut dysbiosis, probiotics, antibiotics, autoimmunity

## Abstract

Myalgic encephalomyelitis (ME) or Chronic Fatigue Syndrome (CFS) is a neglected, debilitating multi-systemic disease without diagnostic marker or therapy. Despite evidence for neurological, immunological, infectious, muscular and endocrine pathophysiological abnormalities, the etiology and a clear pathophysiology remains unclear. The gut microbiome gained much attention in the last decade with manifold implications in health and disease. Here we review the current state of knowledge on the interplay between ME/CFS and the microbiome, to identify potential diagnostic or interventional approaches, and propose areas where further research is needed. We iteratively selected and elaborated on key theories about a correlation between microbiome state and ME/CFS pathology, developing further hypotheses. Based on the literature we hypothesize that antibiotic use throughout life favours an intestinal microbiota composition which might be a risk factor for ME/CFS. Main proposed pathomechanisms include gut dysbiosis, altered gut-brain axis activity, increased gut permeability with concomitant bacterial translocation and reduced levels of short-chain-fatty acids, D-lactic acidosis, an abnormal tryptophan metabolism and low activity of the kynurenine pathway. We review options for microbiome manipulation in ME/CFS patients including probiotic and dietary interventions as well as fecal microbiota transplantations. Beyond increasing gut permeability and bacterial translocation, specific dysbiosis may modify fermentation products, affecting peripheral mitochondria. Considering the gut-brain axis we strongly suspect that the microbiome may contribute to neurocognitive impairments of ME/CFS patients. Further larger studies are needed, above all to clarify whether D-lactic acidosis and early-life antibiotic use may be part of ME/CFS etiology and what role changes in the tryptophan metabolism might play. An association between the gut microbiome and the disease ME/CFS is plausible. As causality remains unclear, we recommend longitudinal studies. Activity levels, bedridden hours and disease progression should be compared to antibiotic exposure, drug intakes and alterations in the composition of the microbiota. The therapeutic potential of fecal microbiota transfer and of targeted dietary interventions should be systematically evaluated.

## 1 Introduction

Myalgic Encephalomyelitis (ME), also known as Chronic Fatigue Syndrome (CFS), is a neglected, serious debilitating disease with no proven diagnostic marker and specific therapy (1, 2). While there is evidence for several pathophysiological abnormalities including the neurological, immunological, infectious, mitochondrial and endocrine system, the underlying etiology and sequence of events remain unknown (3). The gut microbiome in contrast has gained much scientific and public attention over the last decade in different research fields. It is an area which is rapidly expanding due to its complexity and manifold implications in health and disease (4, 5). The evolution of DNA-sequencing methods with reduced costs, reduction of sequencing errors, higher throughput and third generation sequencing has advanced insight into the microbiome (6, 7). The role of the microbiome in ME/CFS has been discussed more and more over the past years, as many patients suffer from gastrointestinal symptoms and there is a very frequent comorbidity with irritable bowel syndrome (IBS) (8) and inflammatory bowel disease (IBD) (9). Juxtaposing the relevance of the immune system in pathogenesis and etiology of ME/CFS (10) with the role of the microbiome in the early development of an ongoing influence on the immune system (11) supports our idea of an interplay between ME/CFS and the microbiome. Increasing recognition of the gut-brain axis in general further motivates closer inspection of this possibility. The aim of this review is to explore and discuss the current state of knowledge on the interplay between ME/CFS and the microbiome (**Table 1**), to identify potential diagnostic or interventional approaches (**Table 2**) and finally to describe areas where further research is needed.

**Table 1:** Overview of results with the theories of possible pathomechanisms concerning the microbiome in ME/CFS.

Mechanism	Question	Findings	Ideas for Future Research
**GUT DYSBIOSIS**	Which role does the gut composition in ME/CFS patients play, can it help to understand the disease pathomechanism and can a specific microbial signature be used for diagnosis?	- Several studies show evidence for intestinal microbiota alterations and dysbiosis in ME/CFS patients (12–16), but results are inconsistent and until now the exact role in the disease mechanism remains unclear (13, 17–19). - In one study researchers were able to classify 83% of the ME/CFS patients correctly based on their dysbiosis in gut and increased inflammatory markers in blood as a consequence of microbial translocation (13).	Larger longitudinal studies with clear clinical criteria and considering different subgroups of patients with ME/CFS to examine if the detected dysbiosis is a cause of the disease or a consequence of patients inactivity, higher use of drugs or history of antibiotic intake. Gut microbiota composition should be assessed at the functional level.
**GUT-BRAIN AXIS**	What role does a gut-brain communication in ME/CFS patients play as it is known that bacteria in the gut produce metabolites which are important for the immune system, hormonal, neural or metabolic pathway to the central nervous system?	- An existing gut-brain communication in ME/CFS patients is supported by different studies showing improvements ○ in neurocognitive symptoms after antimicrobial and probiotic interventions ○ of symptoms after rectal infusions of cultured bacteria (20) ○ of symptoms after antibiotic treatment (21).	Coupled metabolomic-metagenomic studies covering metabolites involved in the gut-brain axis.
**INCREASED GUT PERMEABILITY AND BACTERIAL TRANSLOCATION**	As a leaky gut can trigger inflammatory changes of many chronic diseases, is there also an association with ME/CFS? What is the role of altered butyrate levels in ME/CFS patients as butyrate is associated with energy production, anti-inflammatory function, epithelial barrier functions and better fitness?	- There is evidence for an increased intestinal permeability in ME/CFS patients: ○ Significantly elevated levels of IgA (66,7%) and IgM (40%) against the microbial translocation marker LPS of bacteria in the blood have been found and correlated with the severity of the illness (22). ○ increased bacteria in the blood of ME/CFS patients followed by an exercise test (23). - The endotoxin damage of Gram-negative bacteria and bacterial translocation might result in an activation of the immune response and systemic inflammation (13). - Different research teams reported a reduced abundance of SCFA- (especially butyrate) producing bacteria in ME/CFS patients (12, 13, 15). - Increased fecal SCFA levels have been found in ME/CFS patients (15).	Dietary interventions, alone or coupled with pre-, pro- or synbiotic treatments, are warranted. Low efficacy may conceivably be increased by considering individual baseline. We recommend longitudinal studies ideally with pre-disease states to examine the causality between butyrate levels, medication intake and activity levels of the patients. Serum butyrate levels never have been measured directly in ME/CFS.
**INCREASED** **D-LACTIC ACID THEORY**	Is there a connection between D-lactic acid producing bacteria in the gut of ME/CFS patients and their neurological symptoms, as the clinical presentation of acute D-lactic acidosis is similar?	- Increased fecal colonization of D-lactic acid producing Gram positive bacteria have been found (16) and absolute fecal lactate levels were decreased in ME/CFS patients (15). - Some researchers hypothesize that accumulation of D-lactic acid through an excess bacterial fermentation leads to it’s increase in blood and brain regions, where it causes the neurological symptoms (16, 24). - No improvement in fatigue has been seen by targeting the D-lactic acid producing bacteria with antibiotics and probiotics (25).	D-lactic-acid levels should be directly measured in serum of ME/CFS patients and correlated with symptoms. D-lactic-acid levels should be measured in serum of ME/CFS patients during intervention studies using pre-, pro- and synbiotics and FMT.
**KYNURENINE PRODUCTION INSUFFICIENCY**	What is the role of the enzyme idoleamine-2, 3-dioxygenase (IDO) in ME/CFS, as IDO plays an important role in regulations and suppressing immune activation in chronic infections and tryptophan is known to be metabolized by gut microbiota?	- The symptoms of an increased IDO activity (resulting in a depletion of tryptophan and generation of kynurenine), are similar to some ME/CFS symptoms. An interventional study showed that the mechanism might play a more important role in the initiation of the disease than during the established disease (26). - Genes of IDO isoforms have been found to be mutated. Therefore a different hypothesis is, that mutations in IDO result in the opposite with low kynurenine levels and an accumulation of tryptophan, leading to the typical pathological steady state and clinical presentation of ME/CFS (27).	Investigating variability in serotonin pathway metabolite levels in ME/CFS, and their changes under dietary and pharmaceutical interventions.
**PAST ANTIBIOTIC INTAKE - HYPOTHESIS**	Is there a correlation between antibiotic use and the development of ME/CFS, as the first description of the disease was only after the worldwide use of antibiotics and it is known that antibiotic use in early life disturbs the microbiome and leads to a higher risk of several diseases?	- A study showed that 78% of the tested non-antibiotic drugs inhibited bacterial growth (28). - Antibiotics provoke the risk of D-lactate toxicity, which clinically correlates with the symptoms of ME/CFS (29). - Antibiotics induce ROS and lead to oxidative stress by damaging not only bacterial cells, but also mitochondrial components (30). - However, benefits in some symptoms of ME/CFS patients were also seen after treatment with antibiotics.	Antibiotic intake, in the first years of life but also later life exposure should be evaluated in ME/CFS patients to examine antibiotics as a trigger, pre-existing factor or cause. Longitudinal studies are desperately needed.

**Table 2:** Possible treatment affecting the microbiome in ME/CFS patients.

	Intervention	Question	Findings	Ideas for future research
**1**	**FECAL MICROBIOTA TRANSPLANTATION (FMT)**	What is the current knowledge on FMT in ME/CFS patients, as positive effects have been reported in other diseases?	- A beneficial response in 70% of ME/CFS patients to a rectal infusion of a specific combination of bacteria has been reported, but all patients suffered from gastrointestinal symptoms (20). - Massive improvement, also concerning energy levels of the patients, have been seen in patients undergoing FMT, also comparing to probiotics (31).	We recommend larger studies with more patients and at different stages of the disease undergoing FMT observing the energy level and other symptoms after the treatment.
**2**	**PROBIOTICS**	Are there any positive effects in the symptoms of ME/CFS by manipulating the gut microbiota of the patients?	- Until now, there is no evidence for an amelioration of the core symptoms of ME/CFS patients through the use of probiotics, although significant changes in the gut have been observed in different probiotic studies (32).	In future probiotic studies it would be interesting to take biopsies of the gut instead of taking stool samples, as well as coupling metagenomic and transcriptomic analysis. In particular interventions increasing abundance of SCFA producers should be investigated.
**3**	**FASTING DIETARY INTERVENTIONS**	Are different modes of fasting able to improve symptoms in ME/CFS patients?	- Until now there is no evidence for fasting dietary or fasting mimicking interventions in ME/CFS.	Projection of findings from fasting interventions in other diseases may motivate such trials also in ME/CFS.
**4**	**OTHER POTENTIALLY GUT MICROBIOME MEDIATED APPROACHES**	Is Vitamin B1 and Ginseng supplementation safe and effective in ME/CFS? Are TCM practices such as acupuncture feasible and effective in ME/CFS?	- A double-blind randomized-controlled cross-over study with 40 IBD patients suffering from severe fatigue investigated the effects of a 20-day high dose thiamine (Vitamin B1) supplementation and showed a significant treatment effect regarding fatigue (33) - Oral administration of ginseng has been shown to have anti-fatigue properties in ME/CFS, MS-related fatigue and cancer-related fatigue in several blinded randomized-controlled studies (34–37) - Acupuncture and moxibustion (traditional Chinese medicine practices) to relieve symptoms of fatigue have been extensively investigated, but study quality is partially low (38–40)	We recommend larger scientifically rigorously randomized-controlled studies with more patients and at different stages of the disease observing fatigue symptoms after the treatment.

## 2 What Is ME/CFS?

### 2.1 Case Definition

ME/CFS has a convoluted definition history, creating confusion by changing terminology and classification in the past (41–43). The disease Chronic Fatigue Syndrome (CFS) was first described in 1988 to propose a new name for the chronic Epstein-Barr virus syndrome (44). In 1994, CFS was redefined by the Fukuda Definition with the criteria of at least six months of unexplained chronic fatigue and requiring at least four symptoms out of eight listed (45). The Fukuda Definition criteria are still frequently used in research.

Years before CFS diagnostic criteria were introduced, the first formal description of Myalgic Encephalomyelopathy (ME) appeared in a Lancet editorial in 1956 after an illness outbreak (46, 47). Later the cardinal symptoms of the neuromuscular disease were described as prolonged muscle weakness, neurological dysfunction and the involvement of other body systems. Muscular fatigue occurs after minimal physical exertion and sensory, cognitive and autonomic functions are impaired (41).

In 2003, a more differentiated disease definition was introduced for both diseases with the Canadian Consensus Criteria. They described the mixed condition ME/CFS with a detailed diagnostic scheme with specifications of immunological, neurocognitive and other impairments and a demarcation to psychiatric diseases (48, 49). Subsequently, researchers increasingly considered CFS and ME to be similar diseases and started applying the hybrid diagnosis ME/CFS. Due to inconsistent use of clinical criteria in research studies, an expert group was formed to develop improved diagnostic criteria for CFS and ME, resulting in an International Consensus Criteria definition (41, 50). According to the consensus report that was based on a study with over 2500 patients, waiting six months for a diagnosis was no longer recommended as a requirement. Fatigue as the cardinal symptom was replaced by post-exertional malaise (PEM). Eight symptoms from four areas and a minimum of a 50% reduction from pre-illness activity capacity are required for diagnosis under this definition (50). In 2015, the US National Academy of Medicine (at that time “Institute of Medicine”) recommended, with little success, to change the hybrid ME/CFS into the new name Systemic Exertion Intolerance Disease (1).

However, several researchers note that ME and CFS originally did not share the same disease definition and perhaps should not be conflated (41, 49, 51). On one side, ME describes more clearly impaired individuals and there is not yet highly objective evidence for inflammation in the brain, which makes the term ME confusing (1, 2, 51, 52), and on the other side, the term CFS often results in stigmatization and trivialization, which does not do justice to the serious complaints and impairments (1, 52). Nevertheless, in practice and research, the hybrid ME/CFS has become a predominantly used term due to the overlapping clinical picture (1, 41, 51). We therefore also use ME/CFS in the present work.

### 2.2 Clinical Manifestations and Consequences

ME/CFS is a severe multisystem disease with a high degree of physical disability that can lead to a completely bedridden condition with a high need for care. The disease is characterized by debilitating fatigue with unrefreshing sleep, neurocognitive impairments and flu-like symptoms such as muscle weakness and pain, headaches, sore throat and tender lymph nodes, among others. The malaise and the accompanying symptoms are worsening dramatically after minimal physical, orthostatic and cognitive activity, which is referred to PEM. PEM is the cardinal symptom and mostly comes with a delay of 24 hours after the overexertion leading to a significant reduction (at least 50%) of the patient’s energy and activity level. The recovery period of such an exhaustion usually takes more than 24 hours and can be prolonged to weeks. The course of the disease is fluctuating, the severity of symptoms can change drastically only in a few days. Patients often describe allergies, intolerance in any sort of stimulation such as temperature extremes, light, noise, specific odors or chemicals and simple conversations (2, 45, 50, 53). Patients often suffer from gastrointestinal symptoms, including constipation, diarrhea and intestinal discomfort (53). There is a frequent comorbidity of ME/CFS with irritable bowel syndrome (IBS) ranging from 38% (54) to 42% (12) and up to 92% (8). A trial with 48 patients indicates that over 70% of patients have gastrointestinal symptoms (13). The medication intake against digestive or gastrointestinal problems is over 35% (54). At least 25% of the patients are house- or bedbound (2, 55, 56), representing the moderate and severe forms of ME/CFS (50). According to a large epidemiological study, almost half of the patients are incapable of working (54). A previous study reported 87% as unable to work (56). 48% of patients are not able to pursue any productive activity during their worst periods (54). Severely affected patients describe their limits with restricting their communications and lying in darkness in bed, not being able to shower regularly, or even listen to music or watch TV (57). The illness, which can be as disabling as multiple sclerosis (MS) or systemic lupus erythematosus (SLE) (58), is defined by the WHO in the ICD-10 as a disorder of the nervous system (59). However, taking into account the fact that there still exist different assumptions about the pathogenesis and etiology, it is debated whether this is the right classification (2, 41, 49).

### 2.3 Epidemiology and Burden

The global prevalence of ME/CFS depends on which disease definition is used and ranges widely in the literature, also because of the lack of objective diagnostic tests (2, 53, 60). The National Institute of Health and Care Excellence (NICE) reported an estimated number of 0.2% to 0.4% of the world’s population suffering from ME/CFS (58). A meta-analysis calculated the prevalence globally as high as 0.8% based on clinical assessments and 3.3% based on self-assessments (61). In 2019 Valdez and co-workers confirmed that ME/CFS is not a rare disease estimating the prevalence rate for ME/CFS to be two times higher than the current estimates at 857 per 100’000 individuals (0.86%) using machine learning techniques (62). ME/CFS affects more females than males (2) by a factor of approximately 1.5 to 2 (63).

The economic burden on society is high, with an estimated $7 billion in direct annual costs only in the US (64). Compared to MS or SLE, the costs are around 50% higher for ME/CFS (62). Patients diagnosed with ME/CFS need more health care resources than the rest of the population before their first recorded diagnosis. Patients often consult several doctors for as long as ten years until a diagnosis is made (65). According to a Danish study in 2015, ME/CFS patients have a lower health-related quality of life score (-0.29 in the EQ-5D-3L score) than patients with MS, cancer or stroke (66). A recently published cross-sectional study confirms that patients with ME/CFS are more disabled than patients with MS in almost all areas, including also with regards to employment and income (67).

### 2.4 Trigger and Progression

ME/CFS usually tends to develop in the middle years of life (68), typically with two different onset patterns: a sudden onset that patients can usually remember or a more gradual onset with a slow worsening. A majority of the patients reported having been healthy before, fully functional and with an active social life (54, 69, 70). A cohort study in the United States in 2019 reported that most patients experienced infectious episodes before the disease onset (64%), followed by stressful incidents (39%) and exposure to environmental toxins (20%), from which they never recovered (54). These findings are consistent with earlier studies that also reported the exceptionally high frequency of an infection-associated onset or of co-infections in ME/CFS patients (69, 71, 72). Although several research teams were able to show that a chronic bacterial or viral infection exists in a subset of ME/CFS patients (72–75), there is no clear evidence that infection is the cause of disease or of any pathogen uniquely involved (17). However, some reports of clustered outbreaks of ME/CFS after epidemics (76) raise the question about specific pathogens more frequently triggering ME/CFS (46, 77).

The severity of fatigue and the symptom constellation can change over time (78). The long-term development of the disease varies from patient to patient (69), but crucially a large number of patients (70 – 90%) reported no improvement over time (54). Although a few patients can show a temporary remission (69), a spontaneous full recovery from the disease is extremely rare occurring in only 5% of patients (79).

### 2.5 Diagnosis

Currently, there is no diagnostic test or validated biomarker for ME/CFS available (2, 80), although some results give hope for a future diagnostic biomarker or the use of an immunosignature assay (13, 81–85). The diagnosis is based on the Canadian Consensus Criteria recommended for research studies (1, 50, 53, 78). As mentioned, PEM is the most distinct symptom for differentiating ME/CFS from other diseases with chronic fatigue (53, 70). A meta-analysis reported PEM 10.4 times more common with a ME/CFS diagnosis than in healthy controls (86). Patients often describe these cognitive and physical exacerbations as “crashes” or “collapses” (57). The worsening after physical activity or even normal daily activities also helps to exclude depression or other diseases, because in most other diseases physical activity normally leads to an improvement of fatigue (87, 88). Disease onset with an infection is another important clue to diagnosis (78).

Even though ME/CFS is a common disease, it is often ignored in medical education and research. Various studies show that around 80% of the patients are not diagnosed correctly due to deficits in medical education (53, 89). This is a major challenge not only for the patients, who have to deal with inexperienced care providers, but also for society and the public health system (2, 64).

Some recently published studies suggested that a diagnostic tool may become available in future, as in several cases ME/CFS patients could be reliably distinguished from controls using platforms including nano-electronic blood-based assays (83), CD8+ T cell analysis (81), fecal metagenomic profiles (12) and metabolite analysis (12, 81, 83, 84).

### 2.6 Etiology and Pathophysiology

The underlying etiology or a specific cause of ME/CFS is currently not known. However, over the past years multiple studies have shown elementary evidence of a multi-factorial etiology involving neuro-immuno-endocrinological patterns, metabolic alterations and also genetic components (53, 90–92). Here, we focus on the findings of a dysfunctional immune system, impairments in energy metabolism, genetic predispositions and neurological abnormalities. Finally, a possible role of the gut and its microbiome will be discussed as a current hypothesis.

#### 2.6.1 Dysfunctional Immune System

The role of an acute infection as the most reported onset (54, 69, 71) is not yet fully understood, but there is a lot of evidence to support the hypothesis of a dysfunctional immune mechanism that is followed by fatigue (26, 93) and results in chronic low-grade systemic inflammation (90, 94). Different immune dysregulations are discussed, such as a proinflammatory signature (95–97), dysfunctional CD8+ T-cells (81, 98), decreased T regulatory cells (99) and reduced cytotoxic activity in NK cells (82, 96, 100). In addition to an overreactive immune system (26), Morris et al. detected in ME/CFS patients the production or presence of damage associated molecular patterns (DAMPs) as an epigenetic variation, which can turn an acute infection into a chronic systemic inflammation (90). Recently, researchers at the Stanford University School of Medicine reported results of a nanoelectric blood cell analysis where the immune cells of ME/CFS patients reacted differently to an osmotic stressor than healthy controls, which could explain PEM and might be useful in the future as a possible diagnostic tool (83). Furthermore, investigating 192 ME/CFS patients and 392 controls, a research team using high-throughput methods found 17 cytokines to be significantly abnormal and consistent with the severity, 13 of these being pro-inflammatory. TGF-β, an anti-inflammatory cytokine, was significantly elevated, which was interpreted as a backlash of the body against a long-standing chronic inflammation with which patients struggle (101). This suggests a correlation between inflammation and ME/CFS. However, these findings are controversial (102) and it has not yet been possible to translate these into a diagnostic marker for ME/CFS, as inflammatory markers are altered also in other chronic inflammatory diseases (17). Thus attention must be taken to further test and validate the hypothesis of ME/CFS as a proinflammatory disease, and the relative role of different proinflammatory mechanisms in its etiology and maintenance.

Besides a wide range of ME/CFS associated infectious agents such as human herpesviruses (HHV-6, HHV-7, HHV-8), *Borrelia burgdorferi*, Enterovirus, Lentivirus, Parvovirus, retroviruses, mycoplasma and many more (73, 93, 103), scientists have long debated the association between EBV infection and ME/CFS (17). Up to 37% of patients reported a past symptomatic EBV infection (54, 71, 104). Altered EBV-specific antibodies and a lack of B-cell memory response to EBV have been detected and provide serological evidence of a deficiency of EBV-specific immune response in a subset of ME/CFS patients. These findings might also have potential for a future diagnostic marker (105). One hypothesis of an infectious related pathogenesis is that a virus as a trigger infection interacts with a dysfunctional cellular immunity including T and B cells as well as altered activity of NK cells leading to viral reactivation (10, 94). Morris and colleagues suggested this consequently might cause mitochondrial dysfunction and decreased ATP productions that result in neurocognitive symptoms like fatigue, aching, disturbances of sleep and inactivity, which are all present in ME/CFS patients (94). A longer persistence of these symptoms can further result in stress and more dysregulated viral proteins (10).

ME/CFS as an autoimmune disease (106, 107) and the possible role of the gut in these models have been major subjects of recent research (92, 108). Results from a study in 268 ME/CFS patients at the Berlin Charité revealed that a subset (29%) of patients had elevated levels of autoantibodies against neurotransmitter receptors, supporting the hypothesis of an autoimmune disease (109). The same researchers showed rapid improvements in symptoms in 7 of 10 patients after removing autoantibodies by immunoadsorption (110).

Of interest, in a cohort study of 1309 ME/CFS patients more men reported an initial infection as a trigger and more women prior to ME/CFS suffered from fibromyalgia and immune symptoms such as Raynaud’s phenomenon, drug or metal allergies (75). As more women than men are affected of ME/CFS (2), this sex difference might reflect a gender-differential immune response. A similar distribution is present in many other immunoinflammatory and autoimmune diseases where women are more affected than men (111). A possible reason for the gender-dependent factors in immunity might be sex hormones themselves, alterations in sex-dependent immune cell such as dendritic cells, macrophages, invariant natural killer T cells and CD4+ T cells, the influence of sex hormones on the microbiome, X-chromosome-located immune genes escaping X-inactivation and of course the vast hormonal changes during the menstrual cycle or pregnancy (112). Female reproductive events have been discussed as potentially triggering the onset or at least affecting the course of ME/CFS, as onset mostly occurs during the reproductive years. A study showed a 31-fold higher risk of ME/CFS for women who have been pregnant (113). Chu et al. reported worsening of ME/CFS symptoms in women during pregnancy (42%), menopause (38%) and menstruation (53%), with 8% reporting onset of disease during pregnancy. In contrast, only 11% reported hormone treatments such as contraception or hormone replacement to have any deleterious impact on the disease (54).

#### 2.6.2 Energy Production Impairments and Oxidative Stress

Since energy loss is an important symptom of ME/CFS, various metabolomics research teams focused on the hypothesis that cells of ME/CFS patients might have problems with energy generation, including impaired mitochondrial energy production (91, 94, 114). There is evidence for reduced mitochondrial function in ME/CFS cohorts regardless of disease severity (114). A study by Shungu and co-workers supports the oxidative stress hypothesis by detecting significantly higher lactate in the ventricle of ME/CFS patients. They suggested that an initial infectious process with a cytokine response (IL-1, IL-6, TNF-α, etc.) leads to increased levels of free radicals like NO and reactive oxygen species (ROS), a harmful pro-inflammatory form of oxygen, which can cause oxidative stress in cells. ROS elevate also blood levels of the oxidative stress marker and vasoconstrictor isoprostane. They concluded that the resulting cerebral hypoperfusion leads to an anaerobic metabolism and therefore the higher lactate in cerebrospinal fluid can be explained (115). Armstrong and colleagues collected blood and urine samples from 34 ME/CFS patients and reported further evidence for this hypothesis by finding higher levels of ROS. They also reported an inhibition in the pathway of glycolysis, lower levels of amino acids and suggested from these findings that fatigue in ME/CFS could be caused by this inefficient energy production (116). Glycolytic impairments in severely affected patients were recently confirmed in addition to the mitochondrial dysfunction (114). Similarly, Naviaux and co-workers found strong evidence for a hypometabolic state, reporting significant variations in 20 different metabolites in ME/CFS patients, of which 80% were reduced (84). Decreased levels of the antioxidant vitamin alpha tocopherol (vitamin E) found in studies with ME/CFS patients further support the potential role of increased oxidative stress (117, 118). Germain et al. reported a broad redox imbalance in ME/CFS patients (119). However, in a current study, the authors could not reproduce their findings (120).

Furthermore, abnormal levels of oxidative stress in the post-exercise period have been found, which might explain the typical post-exertional malaise (121). The results of another trial suggested that in ME/CFS patients especially the metabolites which play a role in lipid- and energy metabolism are altered, including increased levels of ceramides, one of the three types of sphingolipids. The elevated plasma levels of ceramides also correlated with increased physical fatigue (122). Focusing more on the “crashes” after minimal exertion, researchers in California carried out a prospective cohort study with 51 participants, where oxygen consumption appeared normal in a first test but was significantly reduced in a second test 24 hours later. With that, the researchers could distinguish the ME/CFS group with an accuracy of 95% (123). Other studies also focused on PEM and showed a possible energy production insufficiency by testing ME/CFS patients in exercises resulting in an increased intramuscular lactate accumulation (124, 125). Chronic immune activation and an increased oxidative and nitrosative stress might lead to an inhibition of mitochondrial function and a defective ATP production, including mitochondrial DNA and electron transport complex damage, reductions of the membrane potential and increase of the mitochondrial permeability, which may play a role in the central metabolic disorders as seen in these patients (94). Another hypothesis is that especially propionate, as a product of gut fermentation, penetrates the intestinal barrier and passes into the blood circulation where it results in oxidative stress and mitochondrial dysfunction, though this metabolite has been more frequently discussed as a health marker. Furthermore, it has been shown that mitochondrial DNA genome variation does not seem to play a role in susceptibility or resistance to ME/CFS, but might affect type and severity of symptoms (126).

#### 2.6.3 Genetic Predisposition

Some studies provide support for the suggestion that genes might play a role in ME/CFS (54), as the disease shows higher concordance in identical monozygotic twins (127). The rates of ME/CFS for first-degree relatives vary from 2.7% (128) to 13% (54). A monozygotic twin study in which more than half (55%) of both identical twin pairs showed ME/CFS symptoms support the evidence of a genetic component, at least in a subgroup of ME/CFS (127). A recent study showed a significantly higher prevalence of the *PTPN22* rs2476601 and *CTLA4* rs3087243 autoimmunity-risk alleles in post-infectious ME/CFS, but not in ME/CFS patients without infection-triggered onset. For the *IRF5* rs3807306 risk allele and the TNF rs1799724 we observed a significantly lower allele frequency in ME/CFS patients without infection-triggered onset (129). Interestingly, these SNPs are associated with enhanced serum levels of TNF-α and IFN-α and are frequently associated with autoimmune diseases of mucosal sites (130–134). It is tempting to speculate that patients who can produce less TNF-α and IFN-α have impaired mucosal immunity and are therefore more prone to develop infection-triggered ME/CFS.

#### 2.6.4 Neurological Abnormalities

There is also evidence from MRI scans for structural and functional brain abnormalities in ME/CFS patients, detected in heterogeneous areas, but mostly related to executive and cognitive functions, memory and perception (135). One trial showed a decreased reticular activation system (RAS) connectivity in the brainstem and hippocampus of ME/CFS patients. They also reported that the extent of the detected deficits correlated with the symptomatology and severity of the disease (136). In another study with 50 patients, EEG abnormalities were detected with signs of a global hypoactivation of the central nervous system. These results suggest an involvement of the central nervous system and might explain the special form of fatigue ME/CFS patients suffer from (137). However, pathological evidence for brain inflammation in ME/CFS patients is limited (2).

### 2.7 Therapy

There is currently no pharmacological or non-pharmacological therapy available with clear evidence for the management of ME/CFS (2, 70, 138, 139). Pacing, the careful dosing of all types of activities as an energy self-management method, is one of the most recommended strategies to avoid relapses due to overexertion. But it is not curative. Patients should learn to read their fluctuating symptoms, pay attention to early signs of PEM and trust their own experience. They have to learn to manage their cognitive, physical, emotional and social activities to reduce a worsening of their symptoms (2, 53, 54, 70, 86). But this is difficult, because the overexertion normally is not recognized immediately and the avoidance of increasing the activity level while feeling well is a major restriction, limiting compliance (57). General treatment should be symptom-based (78), for example wearing eyeshades against light sensitivity or headphones that protect the patient from sound and noise sensitivity (6). Over half of patients use a variety of medications to manage sleep, pain and endocrinological problems and to cope with mood and gastrointestinal issues (54). Among them are also many drugs that are off-label, experimental or non-prescription (57). The new published clinical guideline of NICE warns that therapy should not represent a fixed increase of any kind of activity, because this may lead to a worsening of symptoms (2).

Few treatment modalities targeting autoimmunity have been evaluated in clinical trials in ME/CFS so far showing some evidence for efficacy of rituximab, cyclophosphamide, and IgG in a subset of patients (93, 107). Removing IgG from plasma by immunoadsorption recently showed first evidence of efficacy (110).

## 3 The Role of the Gut Microbiome in ME/CFS

### 3.1 Overview of the Microbiome

In some experts’ perspective, the microbiome with all its genetic information, metabolism, physiology and pathophysiology as well as transplantability should be considered a separate organ (140–142). Considering that the human body contains the same numbers of human and bacterial cells, this is not surprising. Of the overall body weight, about 0.3% is composed of bacteria and the highest density of microorganisms can be found in the digestive tract (143). The term microbiota encompasses the totality of all bacteria and other microorganisms like fungi, protozoa and viruses contained in the body and on the skin (142, 144).. The microbiome is defined as the community of these microorganisms (microbiota) in their well-defined habitat and also refers to their structural elements, metabolites, genetic information, and their surrounding environmental conditions. It is also described as a dynamic interactive micro-ecosystem, which is integrated in the macro-ecosystem where it has crucial functions for health (144). We focus here on the composition and the function of the microbiome in the gut, which is extremely diverse and different in every human, influenced by eating habits, other environmental exposures, sleep, stress, genetic factors and medical treatments (4, 5). Microbial colonization begins immediately after birth, so that the mode of delivery (vaginal or *via* elective Caesarean (C-) section) has an effect on the establishment of the microbiome (145). C-section is accompanied by reduced bacterial diversity in the gut, an altered development of the immune system, and increased allergies and asthma (146). After birth, how an infant is fed (with oral to skin contact during breastfeeding or with more distance as with formula) has profound effects on the early microbial colonization. In addition, breastfeeding affects the neonatal microbiome both directly through the milk microbiota as well as indirectly through shaping of the neonatal immune system through ingredients such as human milk oligosaccharides, secretory IgA, antimicrobial proteins (AMPs) and tryptophan metabolites (147). The composition of microbiota is already relatively adult-like and stable after the first three years of life. Maternal factors, birth mode, the exposure of postnatal factors like antibiotics, breastfeeding and the environment shape or disrupt the ecosystem of the neonatal microbiome in this early phase and can influence the development of the microbiome and the immune system (145).

The gut microbiome as a complex has multiple functions. One important function is the energy extraction from food by fermenting carbohydrates resulting in the production of various compounds for the host (148) like the short-chain fatty acids (SCFAs) butyrate, acetate and propionate (149) and amino acids like γ-aminobutyric acid (GABA) and vitamins (150). Intestinal microorganisms not only play a role in the digestive process and as an important barrier against pathogens (i.e. colonization resistance), but also train the immune system resulting in the production of antibodies and secretion of AMPs. AMPs, toll-like-receptors, innate lymphocytes and IgA-production are all developing in the epithelial cells after gut colonization in a newborn (11). The gut epithelium recognizes signals from the immune cells in the lamina propria and serves as a mediator between intestinal microbiota and the immune system. The recognition of pathogen-associated molecular patterns (PAMPs) of microorganisms, for example lipopolysaccharide (LPS), is ensured by receptors such as TLR, NOD, and PRR (5). Other important functions are metabolism, the production of vitamins, steroid hormones and neurotransmitters (151) and the modulation of mood, cognition and memory through various mechanisms forming a “gut-brain axis” (152, 153). Overstimulation through bacterial dysbiosis, LPS or an increased gut permeability can lead to systemic inflammation (153).

There are beneficial disease-preventing bacteria like Bifidobacteria or Lactobacilli, but also bacteria that are associated with disease development (13, 154). For a long time neglected, studies of the microbiome have recently become more popular with the advent of new molecular methods, efficient computational pipelines and enhanced reference databases to investigate its composition (7, 155). These new methods allow to search for specific disease biomarkers and evaluate treatment options by influencing the microbiome. A large number of studies reported that shifts in the microbiota towards a loss of health-associated bacteria and an accumulation of pathogenic bacteria are associated with metabolic and immune impairments and several disease states including susceptibility to infectious diseases, obesity and metabolic problems, liver diseases, neurological diseases and cancer (5, 156–158). The phyla Firmicutes and Bacteroidetes make up 90% of the gut community, and their ratio has been linked to intestinal and host metabolic health, though not always consistent between studies. Gut ecosystem gene richness (i.e. the number of unique genes) and taxonomic diversity (i.e. the number of bacterial taxa represented) have been strongly linked to gut health and may indicate better resistance to external stress factors. Both measures decrease with age (145, 150, 158). A higher intestinal microbial diversity is also associated with a better host fitness (159), thicker intestinal mucosa and increased production of AMPs. Also, capacity to produce different SCFAs including butyrate and propionate is associated with healthy intestinal composition. For example, in metabolic disorders the production of butyrate and propionate is low (5).

A “perfect” microbiotal state is not recognized, as intestinal homeostasis varies strongly even in health (158, 160, 161). It is also challenging to attribute bacterial markers to single diseases because these trends often overlap and because of the dynamic nature of these findings. Thus some researchers discourage the use of the arguably inconsistent term “dysbiosis” and warn against inappropriate conclusions from research results (5, 157). Despite the need for such caution, repeated findings of microbiome alteration common to diseases sharing pathomechanisms indicate further insights that could be systematically investigated.

### 3.2 Gut Dysbiosis in ME/CFS Patients

Intestinal microbiota alterations and dysbiosis have already been detected in several ME/CFS studies (12–16), but a specific consistent microbial signature has not been found yet (13, 17). The overview of microbiome studies in ME/CFS patients shows an inconsistency of results where an exact link between alterations in the intestinal bacteria and the disease mechanisms cannot be made (18). Newberry and co-workers reported in their systematic literature review that there were eight agreeing and seven conflicting results between ME/CFS microbiome studies, while there is overall evidence for dysbiosis (19).

In one noteworthy study the investigators were able to classify 83% of patients correctly as ME/CFS patients or controls based on their evaluation of dysbiosis in gut microbiome and some increased inflammatory markers in blood as a consequence of microbial translocation, though sample size (N=48) was relatively small and future replication is needed. A reduction in bacterial diversity and richness, lower anti-inflammatory species carriage and higher levels of pro-inflammatory bacterial species such as Enterobacteriaceae were found in a 16S rRNA stool sample analysis (13). Normally, a high microbial diversity is associated with a healthy and fit human host. Cardiorespiratory fitness, which is measured by the maximum oxygen consumption (VO2 peak), maps to about 20% of the variation in gut diversity (159). This means that reduced gut gene richness and microbial diversity in ME/CFS patients also might be associated with a different physical fitness activity. Significantly lower VO2 peaks in ME/CFS patients have been described (162). While Armstrong and co-workers found significant absolute decreases of intestinal bacteria, mainly by the anaerobic bacteria (15), the opposite with an increase of anaerobic microorganisms has also been reported in ME/CFS patients (16).

Higher levels of *Enterococcus* spp (16), *Streptococcus* spp (16, 163), lower levels of the beneficial *Bifidobacteria* (13, 154) and decreased anti-inflammatory Firmicutes (13, 14) have been detected. Enterobacteriaceae were increased in several studies (13, 16, 22). According to the metagenomic analysis by Nagy-Szakal and co-workers, specific bacterial taxa such as the Firmicutes phylum, and the *Faecalibacterium*, *Roseburia* and *Clostridium* genera were associated with ME/CFS. In particular an increase of *Alistipes* and a decrease of butyrate-producer *Faecalibacterium* were considered as top biomarkers with potential diagnostic value (12). Their finding of a higher abundance of the *Clostridium* genus contradicts the opposite results reported by Giloteaux and co-workers (12, 13). The lower abundance of *Faecalibacterium* was also replicated (13). A case report published by the same author compared ME/CFS-discordant monozygotic twins and also identified abundance of *Bifidobacterium* and *Faecalibacterium* to be reduced in the affected twin compared to the non-affected one in addition to lower gut alpha diversity (164). Interestingly, a reduction of *Faecalibacterium* was also found in IBD patients with fatigue (compared to IBD patients without fatigue) (165), cancer-related fatigue (compared to cancer patients with low fatigue) (166) and other autoimmune diseases such as MS (167, 168) and diabetes type 1 (169).

Several studies reported a reduction of a butyrate-producing *Bacteroides* species in ME/CFS (19). High-throughput 16S rRNA sequencing from stool samples revealed gut microbiome alterations that were consistent with an increased inflammation of the gut, namely significantly increased *Lactonifactor* and *Alistipes* (14).

One study with 48 patients and 52 controls identified 26 markers including bacterial taxa that were distinct for ME/CFS and healthy controls, respectively (170). Gut abundances of *Coprobacillus*, of *Eggerthella* and *Blautia* were best able to distinguish patients from controls. Decreases in *Faecalibacterium* (12) and increases in *Coprobacillus* were found and also seen in other previous studies, as discussed above (13). To evaluate the diagnostic potential of the markers, THE researchers separated patients into short-term and long-term duration of ME/CFS. They found that microbiome changes were much more prevalent during the short disease duration (≤ 3 years) compared to a long disease duration (> 3 years). The authors suggest that these microbial markers mirror temporary changes in the early disease stage and hence show potential for future biomarker application (170). Furthermore, a study comparing the microbiome composition of ME/CFS patients to acute Q-fever patients suffering from fatigue as well as healthy controls, found the microbial signature of ME/CFS and Q-fever to be quite similar, while both groups differed profoundly from healthy controls. ME/CFS and Q-fever fatigue syndrome patients further shared the same set of pro-inflammatory markers with which a differentiation of each patient group from healthy controls was possible. The abundance of Firmicutes and Actinobacteria was increase in ME/CFS patients (n = 50) compared to healthy controls (n = 72) while Bacteroidetes were reduced (171).

Another recent study with 35 patients and 70 controls also found a distinct microbial pattern with decreased anti-inflammatory Firmicutes (117), confirming previous results (13, 14). This study compared patients with “internal” (relatives of the patients) vs. external controls and found several overlaps between patients with their relatives, but not with external controls, emphasizing the role of controlling for lifestyle, genetic confounders, and the choice of representative controls (117).

These partially inconsistent but evident findings of dysbiosis in ME/CFS have been described by previous reviews (18, 19), but their exact role in the disease mechanism remains unclear (**Table 1**). The above studies vary strongly with regards to sample size, recruitment (including severity at sampling), and methods for microbiota characterization (most studies using culturing or 16S amplicon sequencing, with only one whole genome shotgun sequencing study to date).

We should take into consideration that ME/CFS patients take many different drugs regularly to manage their variable symptoms (32, 54) and it is known that not only antibiotics, but also other drugs have a significant effect on the commensal microbiota (28, 172, 173), which future work must account for. Diet may also rapidly influence the microbiome in the short term (174). Long-term dietary habits have also been associated with distinctive faecal microbiome genera, e.g., high protein/animal fat diets are associated with *Bacteroides*, whereas diets high in carbohydrates are associated with a high prevalence of *Prevotella* (175). To account for this, dietary intake from at least 48-24 h prior to stool sample collection should be considered. In addition, larger studies with clear clinical inclusion criteria are needed to consider the different subgroups of patients with ME/CFS, as well as consider the viral and eukaryotic components of the gut microbiome (17–19, 176).

### 3.3 The Gut-Brain Axis in ME/CFS Patients

The old sayings to listen to one’s gut feelings, or that something upsets one’s stomach, may not have gained traction by coincidence. Several animal studies provide direct evidence that the intestinal microbiome communicates bidirectionally with the brain (177–179). Signals from the brain have consequences on our intestinal function and vice versa visceral afferents send signals to the brain. These effects can already be seen in daily life, where anxiety, emotional stress or pain influence stool regulation and can lead to diarrhea or obstipation. The routes of communication from the gut to the central nervous system are expected to involve at least 4 pathways, the (I) immune system pathway by cytokines, (II) the hormonal route by the neurotransmitters GABA, serotonin or dopamine, (III) neural routes including the vagus nerve and spinal pathway and (IV) the metabolic pathway including SCFA (180, 181). Neurotransmitters like dopamine or GABA and also signaling molecules are stimulated or generated by intestinal bacteria. Metabolites produced by bacteria are for example SCFAs acetate, butyrate, and propionate (152), and the amino acid and precursor of the neurotransmitter serotonin, tryptophan (182). Some bacteria are also known to produce neurotoxic metabolites (153). The gut-brain communication has been supported by finding signatures of the gut microbiome of increased risk of developing diseases such as post-traumatic stress disorders (183). Here the transplantation of intestinal bacteria from depressive patients to rats induced a typical depressive behavior in the germ-free animals (184). Overall the microbes in the intestine are able to send signals to the brain and influence sleep, stress, memory, mood and cognition (153).

Support for a role of gut-brain communication in ME/CFS has been provided from different fields of studies. Antimicrobial and probiotic interventions in ME/CFS patients provide first evidence for gut-brain communication because of an improvement of different neurocognitive symptoms (163, 185, 186). The D-lactic acid-theory discussed below is also based on the microbiota-gut-brain connection. This hypothesis states that accumulation of D-lactic acid through bacterial fermentation leads to its increase in blood and then in brain regions, where it causes neurological symptoms like fatigue (16, 24). Furthermore the improvement of ME/CFS symptoms after rectal infusions of cultured bacteria (20) and after antibiotic treatment provides evidence for a role played by gut-brain interaction in ME/CFS patients (21). Also in other diseases like depression, treatment with antibiotics suggests a reduction of some symptoms, according to a recently published trial (187). In contrast it should also be noted that antibiotics may also contribute to dysbiosis and disease, especially when used over a long time (188, 189).

### 3.4 Increased Gut Permeability and Bacterial Translocation in ME/CFS Patients

Under normal composition of the gut, bacterial translocation through the gut barrier should not be possible (5). However, there is evidence that intestinal epithelial barrier dysfunction with concomitantly increased permeability, so-called “leaky gut”, is present under inflammatory changes in many diseases such as IBD, IBS, liver diseases, diabetes mellitus and chronic heart failure. Interestingly, in a majority of these diseases there is also an association with gut dysbiosis (190). The subsequent risk for developing ME/CFS in patients with IBD, especially Crohn’s disease, is significantly higher compared to individuals without IBD (3.7% vs. 0.2%) (9). The pathogenesis of the relationship between IBS and ME/CFS remains unclear. One explanation might be that some gut disorders seen in ME/CFS patients such as a dysfunctional gut barrier result in an “irritable” bowel (191). There are also speculations about the common pathophysiology of bacterial translocation (9). The reverse angle was taken by Nagy-Szakal and co-workers who suggested that IBS symptoms might be a consequence of the pathophysiological predisposition or the psychological stress ME/CFS patients have to deal with. In their metagenomic study these authors showed that the signature of the gut microbiome in ME/CFS patients is not fully reducible to an IBS comorbidity. They pointed out that since both syndromes show similar symptoms, some, such as fatigue, might be mistaken for ME/CFS signals and some “ME/CFS bacterial taxa” shown to be typical in different microbiome studies might be linked rather to the IBS comorbidity than to ME/CFS (12). Another possible explanation is the “dysbiotic march hypothesis” from IBS to ME/CFS by Berstad and co-workers. They suggested a three-step progression where excessive use of antibiotics in childhood might lead to typical abdominal symptoms of IBS and, years later, to the development of chronic inflammatory diseases possibly including ME/CFS (192). A similar hypothesis was stated by Kuo et al., who showed a significantly increased risk in women of developing ME/CFS following *Helicobacter pylori* associated peptic ulcer disease in a recent retrospective cohort study (193).

Also several research teams reported an increased intestinal permeability in ME/CFS patients supporting the widespread hypothesis of bacterial translocation into the systemic circulation (6, 9, 23, 97), in order to explain the abnormally increased levels of IgA and IgM against LPS in peripheral blood of ME/CFS patients would not be possible (67% and 40% of ME/CFS patients showed increased IgA levels and IgM levels, respectively, compared to 0% in controls). In addition, IgA levels increased with severity of the illness (22). Furthermore, the elevated levels of bacterial components in plasma of ME/CFS patients indicate increased gut permeability. According to Giloteaux and co-workers it might be possible that increased endotoxin levels damage the epithelial barrier and infiltrate into the bloodstream, where it provokes an immune response and results in systemic inflammation. The reason behind higher levels of the microbial translocation marker LPS in circulation might be the higher intestinal abundance of Gram-negative bacteria as the bacterial endotoxin, which is found in their outer membrane, is associated with gut dysbiosis (5, 13). Nagy-Szakal and co-workers detected in addition elevated levels of plasma ceramide and assumed that this might be a hydrolysis product of increased LPS. The authors speculated that higher ceramides in turn might lead to a toxic reaction in different cell types, including gut epithelial cells, resulting in a dysfunction of the epithelial barrier and increased gut permeability, noticing that higher levels of ceramines have also been found in other chronic diseases such as IBS, diabetes, cardiomyopathy, and atherosclerosis (122).

Another reason for increased intestinal permeability might come from the increased by-products of the amino acid fermentation to produce SCFA (15), as discussed below.

Bacterial translocation could also be important concerning PEM. Shukla and co-workers examined this connection in a small study by observing phenotype characteristics, post-exertion malaise and microbiome changes 15 minutes, 48 and 72 hours after a maximal exercise test. Besides the expected large changes in pain, fatigue and confusion, interestingly also the relative abundance of the intestinal microbiota changed differentially than in the control group. Following exercise, the genus *Clostridium* was increased in the blood fifteen minutes later and abundances of *Bacilli* were significantly higher 48 hours later, which might be due to increased post-exertional bacterial translocation. The authors also pointed out that researchers should rather focus on the post-exertional changes in intestinal composition, than on the dysbiosis itself (23).

Reduced abundance of SCFA producing bacteria, especially those which produce butyrate like *Faecalibacterium* (12, 13) *Roseburia* (Firmicutes) (13) or some *Bacteroides* spp (15)., has been observed by different teams in ME/CFS patients. Serotonin, GABA and SCFAs are produced by bacterial fermentation and are mediators of gut-brain communication. SCFAs such as butyrate are considered the main energy source of colonocytes and are speculated to be a key regulator in the neuro-immunoendocrine system. They are associated with intestinal health by having anti-inflammatory effects and by enhancing intestinal epithelium barrier function (194).

A high-throughput sequencing study revealed that in physically fit humans, high fecal butyrate levels strongly correlated also with extent of physical fitness, therefore also with higher VO_2_ peaks, and interestingly, also with increased gut microbial diversity. Especially higher levels of butyrate-producing organisms were associated with better fitness and higher VO_2_ peaks correlated in the fit individuals with lower LPS biosynthesis. While higher LPS levels in blood normally lead to a strong inflammatory response, lower LPS levels induced by physical activity may explain the reduction of inflammation in fit individuals (159). If we connect these study results from the healthy population with the clinical picture of ME/CFS, a possible explanation for the decreased abundance of butyrate-producing bacteria might be that a reduction of SCFA results in PEM which in turn promotes inactivity, and inactivity in turn could subsequently lead to increased LPS in ME/CFS patients. The inverse scenario may also hold, such as physical inactivity generally follows from ME/CFS symptoms. And this inactivity might lead over time to a reduction of SCFA producing bacteria. A possible third option is that the depleted SCFAs are a secondary effect of the disease. SCFA producing bacteria might for example be repressed by higher levels of D-lactate producing bacteria. Higher levels of D-lactate producing bacteria have previously been reported in ME/CFS patients (16) and possible effects have been explored (25).

In contrast to this butyrate deficiency hypothesis, one study revealed an unexpected significant increase of fecal butyrate, isovalerate and valerate concentrations in ME/CFS samples. These increased fecal SCFA levels correlated with an increased bacterial fermentation. One suggestion by the authors about these altered metabolites was that it might originate from an elevated pH level in the gut or also from gut dysbiosis (15). This finding is also interesting, as SCFAs also can have neurotoxic effects under some circumstances (153). Another possible explanation for higher fecal levels of SCFAs may be an uptake deficiency. Therefore it should be considered that the fecal excretion of SCFA cannot be used as a full reflection of the actual concentration and production of SCFA in the intestine but should be complemented with serum levels (195). However, butyrate itself in serum has never been measured in any study to date. Caution is required in interpreting these results because multiple causal and indirect scenarios are possible. For example, ME/CFS patients are frequently medicated (54) and prevented from physical activity (50), and these two facts might indirectly drive their aberrant intestinal microbiota. Longitudinal studies over a long period, ideally including very early or even better pre-disease states, would be necessary to investigate causality, by assessing the correlation between butyrate levels or dysbiosis in general and the extent of symptoms. These should also account for medication intake and for extent of physical activity as potential confounding factors.

### 3.5 Increased D-Lactic Acid – Theory in ME/CFS Patients

Lactic acid is one of the main by-products of bacterial fermentation, and generates stereoisomers L-lactic acid or D-lactic acid. D-lactic acid is known to cross the blood-brain barrier (196, 197). There were some indications that the clinical presentation of acute D-lactic acidosis is similar to the neurological symptoms of ME/CFS patients and an excess production and absorption of D-lactic acid in blood and brain was proposed as a possible mechanism in ME/CFS (16, 163). Sheedy and co-workers explained the mitochondrial and neurocognitive dysfunction by increased colonization of D-lactic acid producing intestinal Gram-positive bacteria in fecal samples (enterococci and streptococci). They hypothetically assumed that blooms of these bacteria would lead to lower intestinal pH-levels, resulting in a higher gut permeability, and besides the already discussed systemic inflammation, immune activation and oxidative stress might lead to a possible increase of D-lactic acid in blood (16). Based on these findings Wallis and co-workers investigated if a decrease in neuropsychological symptoms could be observed after targeting the overgrowth of the lactic acid producing Gram-positive *Streptococcus* genus with antibiotics and probiotics. By reporting no improvements in fatigue, they raised doubt about the D-lactate theory (163). Interestingly and a major limitation so far for this theory, blood lactate levels have not been measured in ME/CFS patients yet (25), whereas an increase in cerebrospinal fluid (198) and surprisingly a decrease of absolute fecal lactate levels has been found (15). It is important to note that fecal lactate levels also depend on uptake by the host and therefore do not necessarily reflect the actual intestinal lactate metabolism.

We should take into account that D-lactic acidosis is a very rare condition in general (197) whereas ME/CFS is a chronic disease in contrast to e.g. medication-induced episodes of acute lactic acidosis which in general do not cause long-term sequelae. There are also critical voices that see the oxidative stress and D-Lactic acidosis hypothesis as a new hype in the probiotic market. It was pointed out that there has never been proof of negative symptoms due to D-lactic acid producing organisms (196), with the exception being in patients with a short bowel (199). Apart from these patients, the intake of D-lactic acid producing bacteria has been proven safe for human health (196).

### 3.6 Kynurenine Production Insufficiency in ME/CFS Patients

One of the newer research topics about the etiology of ME/CFS patients is an abnormal metabolism of tryptophan to kynurenine including its catalyst indoleamine-2, 3-dioxygenase (IDO) expressed in antigen-presenting cells (26, 27, 200, 201). The precise measurement of tryptophan and its metabolites is an interesting approach for potential biomarkers and therapies in current general research (202). Physiologically, the essential amino acid tryptophan is metabolized approximately 5% to serotonin and melatonin, and degraded approximately 95% by the enzyme IDO to kynurenine (182). IDO and the kynurenine pathway play important roles in the immune system as immunoregulators and immunosuppressors (203). IDO is induced by infection and inflammation through pro-inflammatory cytokines and mediators, mainly by IFN-y, but also IFN-α, TNF-α and LPS (204). Elevated IDO expressions seem to be an important hallmark of chronic viral infections such as HIV, hepatitis B and C by building an immune tolerant environment. Although elevated levels of IDO seem to correlate with the severity of diseases, the relevance of these findings remains unestablished (205). IDO activates regulatory T-cells and suppresses effector T-cell proliferation and function by the degradation of tryptophan to kynurenine (203), which enhances immunologic tolerance towards chronic infections.

A team around Robert Phair recently published an interesting hypothesis about an insufficient kynurenine production in ME/CFS patients, called the “metabolic trap hypothesis”, resulting in an abnormal steady-state of antigen-presenting cells, serotonergic neurons as well as serotonin- and melatonin producing cells. They demonstrated in a small patient population strong support for this theory. The hypothesis implies gene mutations in the isoforms of IDO, which would result in low kynurenine levels and a state with impaired CNS, gastrointestinal, immune and energy metabolism functions. Their examined ME/CFS patients had many more gene mutations in one of the isoforms of IDO, IDO-2, and also the number of mutations correlated with the severity of ME/CFS symptoms. The proposed mathematical model suggested that a lack of the enzyme activity of IDO-2 leads to a stop of the degradation of tryptophan to kynurenine and therefore tryptophan levels will rise in blood and in the cells, which the authors linked to the typical pathological steady-state of ME/CFS. Tryptophan levels in the ME/CFS cells actually were increased and also the expected lower kynurenine concentrations were confirmed. The authors concluded that if tryptophan is increased and the pathway to kynurenine blocked, activity of the serotonin and melatonin pathway will be disrupted. The clinical presentation would fit into this model because serotonin and melatonin dysregulation may both be involved in the symptomatology of ME/CFS. The consequence of the deficient kynurenine production would be among other things an insufficient synthesis of NAD+, which would affect the energy metabolism and potentially explain the hypometabolic phenotype of ME/CFS (27). Furthermore, while low levels have been shown to protect and repair the integrity and permeability injury of intestinal tight junction caused by LPS, elevated levels of tryptophan do not show these properties (206).

Under this “metabolic trap hypothesis”, possible immunological and metabolic mechanisms in ME/CFS are highlighted, with additional implications for the microbiome in ME/CFS patients. On one hand serotonin as a neurotransmitter in the CNS and in the gut wall is known to play a key role as a hormone in the gut-brain crosstalk (207). On the other hand tryptophan is not only metabolized through the endogenous serotonin, kynurenine and melatonin pathway, but also by the gut microbiota into indole derivatives (208). This means that changes in the function of IDO also have effects on the fermentation of tryptophan and the microbiome. A reduced activity of IDO is known to lead to an impaired intestinal mucosal barrier, increased endotoxin translocation and chronic inflammation (209), all things we have discussed to be present in the disease.

In contrast, there also exist thoughts in the opposite direction of the “metabolic trap hypothesis”, namely about an increased kynurenine production in ME/CFS patients, instead of a decrease. The depletion of tryptophan and generation of kynurenine through an increased IDO activity resulting from an activated immune response is expressed by the kynurenine and tryptophan (KYN/TRP) ratio, which is therefore a marker for the IDO activity and for cellular immune response in *in vitro* studies (210, 211). The fact that high IDO levels are associated with various chronic inflammatory settings (209), might support the thought of an increased kynurenine production. Also, the KYN/TRP-ratio and some of the kynurenine pathway metabolites such as the neuroactive and excitotoxic quinolinic acid have been observed to be higher in many diseases (212) and symptoms overlap with ME/CFS, such as hyperalgesia or higher sensitivity to pain, light, sound or touch, called central sensitization (26). Additionally, EBV infection, a common trigger of ME/CFS, induces IDO to degrade tryptophan to kynurenine (213). Along another line of reasoning, tryptophan metabolism has also been examined in IBD and IBS, which both have been associated with ME/CFS (32, 54). Changes in tryptophan metabolism have been shown to be involved in IBD, revealing lower serum tryptophan concentrations and bacterial tryptophan metabolites as well as increased kynurenine and higher IDO-1 activity (214). These findings suggest that tryptophan and its metabolites might have an important effect on the gut microbiome, the gut mucosal immune activation and its interaction with the host such as in the immune response (208, 214, 215). A recent interventional clinical study investigated such a high kynurenine hypothesis, and they revealed no direct alteration of KYN/TRP ratio in diagnosed ME/CFS patients. The researchers concluded that an abnormal immune activation and kynurenine metabolism might play an important role in the initiation of the active fatigue response of ME/CFS, but probably not later during the established syndrome (26).

### 3.7 Past Antibiotic Intake Hypothesis in ME/CFS Patients

It is well known that the postnatal and early life use of antibiotics disrupts the neonatal microbiome and is associated with a higher risk of several diseases such as asthma, IBD or type 2 diabetes (145). Also in adults there is, for example, an increased risk of 13% to develop diabetes after an antibiotic treatment (216), which supports that changes in the microbiome alter some metabolic conditions. According to a very large retrospective study, one treatment with antibiotics also increases the risk of developing IBD by 84% and with every following treatment there is a 6% higher risk. The researchers were also able to show that the earlier and more frequently antibiotics were taken, the higher was the risk of IBD (217). IBD is also often mentioned in connection with ME/CFS, although the correlation remains unclear (9, 13). Interestingly, the mode of delivery influences the amount of antibiotic resistance genes in the microbiome, with vaginally delivered infants appearing to be relatively protected against antibiotic resistance (150). Berstad and co-workers suggested with their three-set “dysbiotic march hypothesis” an explanation for the high comorbidity in ME/CFS patients with IBS, as described above (192).

After the discovery of penicillin, antibiotics massively intruded into medical practice in the 1940s and 1960s (218). According to a recent report, antibiotics disrupt the bacterial composition in the gut for up to 4 years and to some extent permanently (145, 219, 220).

Interestingly, the altered taxa by antibiotics (219) overlap with some altered taxa in ME/CFS patients shown by Nagy-Szakal and co-workers (12). To our knowledge, surprisingly, there is currently no study that focused on the antibiotic intake as a cause, trigger or pre-existing factor for developing ME/CFS. As described above, patients with ME/CFS tend to have previously suffered from frequent infections and there is plenty of evidence for an infectious trigger, proven by history, microbial or antigen detection (54, 71, 72, 92, 104). This in turn is likely linked to a higher antibiotic use in these patients as well. As Kronman and co-workers also noted, this does not necessarily mean that children with more frequent infections and antibiotic intake are more vulnerable to IBD or ME/CFS. However, a possible mechanism could be that antibiotic use changed the microbiome in such a way that their intestinal microbiota became more susceptible to some infectious or non-infectious triggers for ME/CFS, for example by impaired capacity to produce protective anti-inflammatory metabolites or increased potential for extraintestinal infiltration (217). More medications than only primary/so-called antibiotics may still possess antibiotic activity; a previous study with 156 tested non-antibiotic common drugs showed that as many as 78% inhibited bacterial growth (28). It is known that antibiotics provoke the risk of D-lactate toxicity. A potential reason for this might be that antibiotic exposure leads to an increase of D-lactate-producing bacteria (29). This hypothesis would be congruent with the hypothesis of a D-lactic acidosis in ME/CFS patients (15, 198) and higher abundance of D-lactic acid-producing bacteria (16).

Interestingly, it might be possible to link the antibiotic intake to the gender imbalance observed in ME/CFS patients. Women are diagnosed with ME/CFS up to four times as often than men (221). A metaanalysis and systematic review showed that women have in general a 27% higher antibiotic exposure than men (reflecting, for example, treatment of urinary tract infections). In the age group of 16 - 54 years the difference was even higher with 40% more prescribed antibiotics for females (222). Also, the mitochondrial and oxidative stress-hypothesis could be explained by previous antibiotic intake, as one study showed that antibiotics induced ROS and led to oxidative damage in human cell lines and mice. According to this study it might be possible that some antibiotics not only target bacterial components, but also mitochondrial components (30). In ME/CFS patients there is evidence for higher levels of ROS (115, 116, 121).

In contrast to this hypothesis, antibiotics have been discussed as possible treatment for ME/CFS, with the aim to reduce the overgrowth of some species (21, 163). Jackson and colleagues revealed in a pilot study sleep improvement after antibiotic treatment, mainly due to a decrease in lactic acid producing bacteria. They assumed that sleep quality might be improved due to a more equilibrated microbiome with reduced levels of Gram-positive bacteria, leading to a reduction of proinflammatory cytokines (21). With an antibiotic and probiotic treatment over 4 weeks Wallis and colleagues could show some benefits in neurological symptoms including sleep, but not fatigue itself (163).

### 3.8 Possible Microbiome Therapies in ME/CFS Patients

Although multiple studies show evidence that inflammation in the gut as well as several diseases are associated with an altered gut-brain communication, evidence for a successful therapy in response to these complaints is rare (180). Two widely discussed therapies are probiotic interventions (95, 185, 186, 223, 224) and fecal microbiota transplantation (FMT) (225–228).

#### 3.8.1 Probiotic Interventions

The idea of changing the intestinal microbiota, enhancing health and preventing diseases by the intake of beneficial live microorganisms sounds obvious and has not only attracted the attention of researchers and gastroenterologists, but also industry and marketing experts (196). A meta-analysis with 1793 patients came to the conclusion that probiotic use is effective for pain and symptom severity management in IBS patients (223). In contrast, a systematic review over all published probiotic meta-analyses concluded that a benefit of probiotics was only evident for antibiotic- and *Clostridium difficile*-associated diarrhea and respiratory tract infections (224).

Two systematic literature reviews examined the usefulness of probiotic treatment in ME/CFS and both only included two studies due to inconsistent outcome measures, poor study quality and limited available studies (32, 229). In a small cohort significant increases of *Bifidobacteria* (73.7% vs. 37.5%) and *Lactobacillus* (73.7% vs. 43.8%) compared to the placebo group were shown after the intake of probiotics (185). Groeger and co-workers showed in one of the rare RCT studies in this field a decrease of several systemic inflammatory markers like CRP or TNF-α in ME/CFS patients (71% vs. 11%) after an oral intake of *Bifidobacterium infantis* (95). Sullivan and co-workers provided some support for the gut-brain communication by observing significant ameliorations in anxiety of ME/CFS patients by probiotic interventions containing *Lactobacillus* and *Bifidobacterium*, but considerate is of note that this is not one of the core symptoms of ME/CFS and that the study enrolled only 15 subjects (186). Another non-controlled pilot study with 13 ME/CFS patients showed improvements in well-being, oxidative and inflammatory parameters after probiotic intake (230).

Although these studies show the potential of the microbiome as a target, a direct and long-term improvement in the main symptom PEM and in physical activity levels has not been proven yet. The systematic review by Corbitt and colleagues summarized the current lack of evidence for the use of probiotics as a treatment of gastrointestinal symptoms in ME/CFS patients, primarily due to the overall poor study quality (32). This conclusion conforms with an evaluation published in Nature that there is still a lack of impact assessment, evidence, and approved therapy recommendations for the use of probiotics in general, as there are still too many conflicting, misleading and ambivalent results and conclusions with heterogeneous studies often pushed by the industry (231). Additionally, it should be considered that all these ME/CFS probiotic studies were conducted with stool samples, but recent findings suggested rather taking biopsies to yield a sufficient picture of the gut interaction and gut health in general, as the site of action in the small intestine may exhibit different patterns. It is also recommended to do a before-and-after analysis of the gut microbiota with regards to the highly individually specific and in some regards resistant gut microbiota (232). In other neuroimmunological diseases, such as MS, a recent review concluded that probiotics modulate the immune system, respectively upregulating Treg cells, anti-inflammatory cytokines and decreasing inflammatory monocytes (233). One of the studies reviewed showed probiotic supplementation decreased dysbiosis associated taxa and to increase the abundance of *Lactobacillus* and *Bifidobacterium* (234). A relatively new field is the investigation of supplementation of SCFA. SCFA producing bacterial taxa have often been found to be depleted in patients, reflecting a dysbiotic state and potentially causing an inflammatory environment relevant in neuroimmunological diseases (235, 236). In a clinical study with MS patients, a propionic acid supplementation was associated with increased Treg cells and IL-10 levels (237), which may have implications for ME/CFS as well. Similar results were found *in vitro* and in animal studies before (238, 239). Clinical studies on the efficacy of probiotics in ME/CFS patients are currently running, e.g. NCT 04741841 and NCT03773003.

#### 3.8.2 Fecal Microbiota Transplantation in ME/CFS Patients

FMT as an alternative therapeutic option has recently achieved broad attention. To date the therapy of antibiotic associated *C. difficile* colitis with oral, enteral or rectal infusion of liquid feces from a healthy donor is the most effective FMT indication with cure rates of up to 90% (225). Other albeit less impressive success has been reported in patients with ulcerative colitis (240), IBS (226), patients with metabolic syndrome, where bacteriotherapy increased insulin sensitivity (241) and as an accompanying measures in patients receiving antibiotics (232). Also correlation between the gut composition and the symptoms of Autism Spectrum Disorders are widely discussed (227). Recently stool transplants from autism spectrum disorder patients to aseptic mice revealed the typical symptoms of autistic behavior in mice, such as social impairments and stereotypies alongside changes in brain gene expressions (242). FMT from healthy donors was successfully applied in a small uncontrolled open-label study to 18 children with autistic disorders who improved after a short time. In a follow-up two years later their gastrointestinal complaints decreased by 58% (227).

In the research field of ME/CFS, fecal transplantation has been discussed, as there are many preliminary indications that the microbiome might be an important player in the disease, especially its neurological symptoms. Undergoing a transcolonoscopic infusion of a combination of Bacteroidetes, Clostridia and *E. coli*, 70% (42 of 60) of ME/CFS patients who suffered from gastrointestinal symptoms had a beneficial transient response to the rectal infusion and 58% (35 of 60) even reached a lasting response. These results should be treated with caution, as the bacteriotherapy was only tested in a subgroup of ME/CFS patients suffering from IBS (52 of 60) (20). Kenyon and colleagues compared in a retrospective Observational Outcomes Study 42 ME/CFS patients with IBS the intake of different nutritional remedies including pre- and probiotics versus ten FMTs over ten days. They evaluated the results by clinical assessment and on a percentage basis from no improvement (0%) to a maximum improvement (100%). Of the 21 patients who underwent an FMT, 17 patients showed a massive improvement of 65% - 95%. The improvement was also significantly higher than in the oral treatment group, also regarding their energy levels. Seven of the fecal transplanted patients were subsequently reported cured with an energy level returning to normal, practically normal or almost normal; some of them suffered for decades from ME/CFS (31). Further, data from two double-blind randomized-controlled trials showed significant improvements in fatigue symptoms in IBS patients partly in a dose-dependent manner (243–245). Interestingly, the FMT increased fecal SCFA levels in IBS patients in one of the studies and the increase was inversely correlated with fatigue symptoms, suggesting that SCFAs might play a role in the pathophysiology (246). These results suggest that FMT might be a possible treatment for at least a subgroup in ME/CFS patients (31). Two double-blind randomized controlled studies aiming to investigate the efficacy of FMT in ME/CFS are currently running (NCT04158427 and NCT03691987).

#### 3.8.3 Fasting Dietary Interventions

Many ME/CFS patients suffer from food hypersensitivity (247). Many patients reported to have tried changes in diet and over 50% believed that the modification reduced their fatigue (248), although there are no specific dietary recommendations or exclusion diets in ME/CFS (58). ME/CFS fasting and nutrition studies are in general limited and rare (249, 250), but the success from other chronic diseases (251) is raising hope that ME/CFS symptoms such as fatigue might also improve with adapting nutrition, fasting or special diets.

An interventional fasting study recently reported success in altering the gut microbiome, reducing blood pressure and body weight by immune modulation in metabolic syndrome patients (252). Fasting diets or fasting mimicking diets have been shown to have an impact on the immune system, potentially improving chronic inflammation (253) without impairing the host defense against viral and parasitic infections (254). Accumulating evidence suggests that these dietary regimens have the potential to ameliorate hyperinflammation *via* inhibiting NLRP3 inflammasome (255), modulating type 2 immune response (256), decreasing intestinal pro-inflammatory Th-17 cells *via* the gut microbiome (257) or showing anti-inflammatory properties *via* other pathways (258).

Impaired immune cell mitochondria and the microbiome were recently suggested to interact with circadian rhythm and driving ME/CFS pathophysiology (259, 260).

As mitochondrial dysfunctions are widely discussed to play a key role in the pathophysiology of ME/CFS patients (91, 94, 114), calls have therefore been made for caloric restriction, fasting and ketogenic diet interventions with the aim to protect the mitochondria, which might reduce some ME/CFS symptoms (261). Mitochondrial ATP production was shown to be inadequate in ME/CFS patients (124, 262). Ketone bodies, as produced during fasting and ketogenic diets, have been shown to improve mitochondrial metabolism, enhance mitogenesis and lower post-exercise lactate concentration in mice (263–265). Ketogenic diets are fasting mimicking diets that can further bypass glycolysis, hence glycolytic impairments that are suspected to be present in ME/CFS, and may provide another main energy substrate for the cell (266). Small partly non-controlled clinical pilot studies have investigated the potential of ketogenic diets in improving fatigue symptoms in multiple sclerosis (267), Parkinson´s disease (268) as well as short-term fasting in cancer-related fatigue (269). A risk of bias should be considered, as dietary studies cannot be blinded. Further, radical dietary regimens should be administered with caution and under guidance of trained medical specialists as it is not clear whether they can cause a worsening of symptoms in ME/CFS patients, especially in the first weeks. While the precise mechanisms of action of ketogenic diets are not completely understood, effects are thought to be at least partially mediated *via* the gut microbiome (257, 270–272).

One systematic review came to the conclusion that there was no evidence for a benefit of elimination or modified diets in ME/CFS patients (250). This included nutritional supplements, which might bring benefits for some symptoms suffered by ME/CFS patients (249, 250, 273).

#### 3.8.4 Other Potentially Gut Microbiome Mediated Approaches

A double-blind randomized-controlled cross-over study with 40 IBD patients suffering from severe fatigue investigated the effects of a 20-day high dose thiamine (Vitamin B1) supplementation. A significant treatment effect was shown, measured with the IBD-fatigue questionnaire (33). Thiamine, an important co-factor in the TCA cycle and involved in glycolysis (274), is partly produced by gut bacteria such as *Bacteroides*, which were shown to be reduced in ME/CFS. *Faecalibacterium* that was also shown to be reduced in many ME/CFS patients (12, 13, 19) requires thiamine for growth (275). Even though microbial production of thiamine only supplies low amounts, availability of thiamine may shape the highly competitive microbial environment (276).

In the light of the absence of therapy options, besides dietary and pharmaceutical therapies, other non-pharmaceutical treatment options have been investigated. Especially in China, the potential of acupuncture and moxibustion (traditional Chinese medicine practices) to relieve symptoms of fatigue have been extensively investigated for several years (38–40). Recently, a randomized controlled trial has been conducted and has shown improved fatigue in ME/CFS patients, associated with alterations in the intestinal microbiome (277). However, studies with these treatment regimens cannot be blinded and the combination with questionnaire-based outcome parameters such as subjectively perceived fatigue scores, cause a high risk of bias. Another non-pharmacological approach is the oral administration of ginseng that has been shown to have anti-fatigue properties in ME/CFS, cancer-related fatigue and MS-related fatigue in several blinded randomized-controlled studies (34–37). Just recently, it was suggested that the related pharmacokinetics are mediated *via* the gut microbiome (278, 279). While these indications are all indirect and thus cannot be considered conclusive, even though randomized controlled trials are considered relatively strong evidence, they support the general principle of effective fatigue reduction accompanying changes to the gut microbiome in similar conditions, which makes assessment of whether the same applies in the ME/CFS setting relevant to test further.

## 4 Conclusion

An association has been shown between the gut microbiome and ME/CFS while there is no evidence of causality. An altered microbiome might play an important role in ME/CFS due to increased gut permeability leading to bacterial translocation and due to fermentation products that directly or indirectly affect various cells, their mitochondria, and the local and systemic immune state. With the concept of the gut-brain axis we see an important possibility that the intestinal microbiome may be involved in the neurocognitive impairments of the patients. The potential diagnostic use of fecal metagenomic and plasma metabolomic analyses, as well as the potential therapeutic use of FMT, should be evaluated in future studies in patients with ME/CFS. The uncontrolled use of probiotics should be reevaluated carefully due the lack of evidence, heterogeneity of studies and quality of products, and considering the marketing hype until better data become available.

Dysbiosis in the gut of ME/CFS patients has been confirmed by many researchers and is evident, but these discoveries as yet may have less value as a possible pathomechanism of the disease as long as causality has not been confirmed. It still needs to be resolved if the altered gut microbiome is a risk factor or a consequence of the disease or merely related to the inactivity and frequent antibiotic use in these patients. Thus far a causal link with specific disease pathogens could not yet be demonstrated in any microbiome studies using 16s-RNA sequencing (187). To move the field forward, we recommend longitudinal studies, where confounders such as physical activity levels and antibiotic intake should be documented ideally prior to disease onset. Rather dysbiosis might be explored for possible diagnostic tools and for guiding research into future therapy possibilities. The role of antibiotics and other medications throughout life, including intrauterine and early childhood use, and reproductive life events such as pregnancy should be systematically investigated as risk or -less likely- protective factors in the development of ME/CFS.

Direct comparisons between ME/CFS microbiome studies are challenging and reveal conflicting results, especially regarding gut dysbiosis. This may be related to different diagnostic criteria being used across studies, resulting in study populations where different neurological, immunological, infectious, muscular and endocrine abnormalities are salient. Similarly, the existence of subtypes of ME/CFS should be considered more, most importantly differences in post-infectious ME/CFS compared to other triggers, but also the highly variable appearance of some symptoms and severity (43, 119). There is broad consensus of a need for better study designs including a consistent use of the case definition in research, a higher study quality as well as more longitudinal studies (13, 14, 22, 134, 163, 232). These points should be considered in future investigations.

## Author Contributions

All the authors contributed to the work presented in this manuscript. CK, PV, RK, and WA planned and conceived the work. RK, LB, and UL performed the literature search and wrote the draft and final manuscript. CS, CK, PV, SF, and WA critically reviewed the drafts and provided input for the final manuscript. CS and SF supervised the process. The manuscript was revised and fully completed by SF, RK, LB, and UL. All authors read and approved the final version of this manuscript and approved it for publication.

## Funding

UL was supported by the German Federal Ministry of Education and Research (EMBARK; 01KI1909B) under the frame of JPI AMR (EMBARK; JPIAMR2019-109).

## Conflict of Interest

The authors declare that the research was conducted in the absence of any commercial or financial relationships that could be construed as a potential conflict of interest.

CS received clinical study grant and speaker honoraria from Baxalta and Fresenius and is consultant for Celltrend.

## Publisher’s Note

All claims expressed in this article are solely those of the authors and do not necessarily represent those of their affiliated organizations, or those of the publisher, the editors and the reviewers. Any product that may be evaluated in this article, or claim that may be made by its manufacturer, is not guaranteed or endorsed by the publisher.
