# Erratum: The Gut Microbiome in Myalgic Encephalomyelitis (ME)/Chronic Fatigue Syndrome (CFS)

**DOI:** 10.3389/fimmu.2022.878196

**Published:** 2022-03-30

**Authors:** Frontiers Production Office

**Affiliations:** Frontiers Media SA, Lausanne, Switzerland

**Keywords:** ME/CFS, Chronic Fatigue Syndrome (CFS), Myalgic Encephalomyelitis (ME), microbiome, gut dysbiosis, probiotics, antibiotics, autoimmunity

Due to a production error, there was a mistake in **Table 1** as published. During the final editing of the table, a formatting error occurred. The publisher apologizes for this mistake.

**Table 1:** Overview of Results with the theories of possible pathomechanisms concerning the microbiome in ME/CFS.

Mechanism	Question	Findings	Ideas for future research
GUT DYSBIOSIS	Which role does the gut composition in ME/CFS patients play, can it help to understand the disease pathomechanism and can a specific microbial signature be used for diagnosis?	-Several studies show evidence for intestinal microbiota alterations and Sdysbiosis in ME/CFS patients (12–16) but results are inconsistent and until now the exact role in the disease mechanism remains unclear (13, 17–19). -In one study researchers were able to classify 83% of the ME/CFS patients correctly based on their dysbiosis in gut and increased inflammatory markers in blood as a consequence of microbial translocation (13).	Larger longitudinal studies with clear clinical criteria and considering different subgroups of patients with ME/CFS to examine if the detected dysbiosis is a cause of the disease or a consequence of patients inactivity, higher use of drugs or history of antibiotic intake. Gut microbiota composition should be assessed at the functional level.
GUT-BRAIN AXIS	What role does a gut-brain communication in ME/CFS patients play as it is known that bacteria in the gut produce metabolites which are important for the immune system, hormonal, neural or metabolic pathway to the central nervous system?	-An existing gut-brain communication in ME/CFS patients is supported by different studies showing improvements •in neurocognitive symptoms after antimicrobial and probiotic interventions •of symptoms after rectal infusions of cultured bacteria (20) •of symptoms after antibiotic treatment (21).	Coupled metabolomic-metagenomic studies covering metabolites involved in the gut-brain axis.
INCREASED GUT PERMEABILITY AND BACTERIAL TRANSLOCATION	As a leaky gut can trigger inflammatory changes of many chronic diseases, is there also an association with ME/CFS? What is the role of altered butyrate levels in ME/CFS patients as butyrate is associated with energy production, anti-inflammatory function, epithelial barrier functions and better fitness?	-There is evidence for an increased intestinal permeability in ME/CFS patients: •Significantly elevated levels of IgA (66,7%) and IgM (40%) against the microbial translocation marker LPS of bacteria in the blood have been found and correlated with the severity of the illness (22). •increased bacteria in the blood of ME/CFS patients followed by an exercise test (23). -The endotoxin damage of Gram-negative bacteria and bacterial translocation might result in an activation of the immune response and systemic inflammation (13). -Different research teams reported a reduced abundance of SCFA- (especially butyrate) producing bacteria in ME/CFS patients (12, 13, 15). -Increased fecal SCFA levels have been found in ME/CFS patients (15).	Dietary interventions, alone or coupled with pre-, pro- or treatments, are warranted. Low efficacy may conceivably be increased by considering individual baseline. We recommend longitudinal studies ideally with pre- disease states to examine the causality between butyrate levels, medication intake and activity levels of the patients. Serum butyrate levels never have been measured directly in ME/CFS.
INCREASED D-LACTIC ACID THEORY	Is there a connection between D-lactic acid producing bacteria in the gut of ME/CFS patients and their neurological symptoms, as the clinical presentation of acute D-lactic acidosis is similar?	-Increased fecal colonization of D-lactic acid producing Gram positive bacteria have been found (16) and absolute fecal lactate levels were decreased in ME/CFS patients (15). -Some researchers hypothesize that accumulation of D-lactic acid through an excess bacterial fermentation leads to it’s increased in blood and brain regions, where it causes the neurological symptoms (16, 24). -No improvement in fatigue has been seen by targeting the D-lactic acid producing bacteria with antibiotics and probiotics (25)	D-lactic-acid levels should be directly measured in serum of ME/CFS patients and correlated with symptoms. D-lactic-acid levels should be measured in serum of ME/CFS patients during intervention studies using pre-, pro- and synbiotics and FMT.
KYNURENINE PRODUCTION INSUFFICIENCY	What is the role of the enzyme idoleamine-2, 3 -dioxygenase (IDO) in ME/CFS, as IDO plays an important role in regulations and suppressing immune activation in chronic infections and tryptophan is known to be metabolized by gut microbiota?	-The symptoms of an increased IDO activity (resulting in a depletion of tryptophan and generation of kynurenine), are similar to some ME/CFS symptoms. An interventional study showed that the mechanism might play a more important role in the initiation of the disease than during the established disease (26). -Genes of IDO isoforms have been found to be mutated. Therefore a different hypothesis is, that mutations in IDO result in the opposite with low kynurenine levels and an accumulation of tryptophan, leading to the typical pathological steady rate and clinical presentation of ME/CFS (27).	Investigating variability in serotonin pathway metabolite levels in ME/CFS, and their changes under dietary and pharmaceutical interventions.
PAST ANTIBIOTIC INTAKE-HYPOTHESIS	Is there a correlation between antibiotic use and the development of ME/CFS, as the first description of the disease was only after the worldwide use of antibiotics and it is known that antibiotic use in early life disturbs the microbiome and leads to a higher risk of several diseases?	-A study showed that 78% of the tested non-antibiotic drugs inhibited bacterial growth (28). -Antibiotics provoke the risk of D-lactate toxicity, which clinically correlates with the symptoms of ME/CFS (29). -Antibiotics induce ROS and lead to oxidative stress by damaging not only bacterial cells, but also mitochondrial components (30). -However, benefits in some symptoms of ME/CFS patients were also seen after treatment with antibiotics.	Antibiotic intake, in the first years of life but also later life exposure should be evaluated in ME/CFS patients to examine antibiotics as a trigger, pre-existing factor or cause. Longitudinal studies are desperately needed.

The original version of this article has been updated.

